# Prognostic significance of AMPK in human malignancies: A meta-analysis

**DOI:** 10.18632/oncotarget.12405

**Published:** 2016-10-03

**Authors:** Ji Cheng, Xiaoming Shuai, Jinbo Gao, Ming Cai, Guobin Wang, Kaixiong Tao

**Affiliations:** ^1^ Department of Gastrointestinal Surgery, Union Hospital, Tongji Medical College, Huazhong University of Science and Technology, Wuhan, China

**Keywords:** AMPK, prognosis, cancer, meta-analysis, malignancies

## Abstract

**Background:**

AMPK is a well-investigated kinase mediating cellular metabolism and stress responses. However, its indicative role in survival prognosis remains ill-defined. Therefore we performed this meta-analysis in order to clarify the prognostic impact of AMPK expression in human malignancies.

**Methods:**

Literatures were retrieved via searching databases of PubMed, Web of Science, Embase and Cochrane Library. Studies comparing the prognostic significance between different AMPK levels among human malignancies were included into the pooled analysis. The statistical procedures were conducted by Review Manager 5.3 and the effect size was displayed by model of odds ratio. Subgroup analyses were additionally implemented to disclose the potential confounding elements. The outcome stability was examined by sensitivity analysis, and both Begg's test and Egger's test were utilized to detect the publication bias across the included studies.

**Results:**

21 retrospective cohorts were eventually obtained with a total sample-size of 9987 participants. Patients with higher AMPK expression had better outcomes of 3-year overall survival (P<0.0001), 5-year overall survival (P<0.0001), 10-year overall survival (P<0.0001), 3-year disease free survival (P<0.0001), 5-year disease free survival (P=0.002) and 10-year disease free survival (P=0.0004). Moreover, the majority of subgroup results also verified the favorably prognostic significance of AMPK over-expression. The outcome stability was confirmed by sensitivity analysis. Results of Begg's (P=0.76) and Egger's test (P=0.09) suggested that there was no publication bias within the included trials.

**Conclusions:**

Higher expression of AMPK significantly indicates better prognosis in human malignancies.

## INTRODUCTION

AMPK, short for AMP-activated kinase protein, serves as a highly conserved metabolic sensor in various tissue types. It is structurally constituted by a catalytic subunit α and two regulatory subunits β and γ in mammals [1]. Decreased level of cellular ATP or glucose deprivation could directly lead to activation of AMPK heterotrimer, which subsequently triggers downstream signaling cascade to enhance catabolic reactions and maintain energy homeostasis [2].

Due to its essential efficacy in metabolic modulation, potential role of AMPK in human tumorigenesis attracts numerous academic attentions. It is currently acknowledged that once activated, the AMPK complex involves in a variety of neoplastic pathways, functioning as a core tumor suppressor [3]. Along with the phosphorylation on AMPKα, its downstream target COX-2 could be greatly depressed, which results in decreased level of inflammatory factors and carcinogenic risk [4, 5]. On the other hand, activated AMPK is able to upregulate the expression of ULK1, which is a positive regulator of autophagy that protects the stressful cells from oncogenic accumulation [6, 7]. Additionally, cellular senescence induced by activation of AMPK-p53 axis is another explanation of the tumor suppressor role of AMPK [8, 9]. Thus based on such mechanisms, AMPK has been confirmed as a vital anti-tumor effector among multiple malignancies including hepatocellular carcinoma [10], colorectal cancer [11], lung cancer [12] and thyroid cancer [13], emerging as a potential therapeutic target in cancer treatment.

However, despite of the accumulating laboratory evidences, the clinical significance of AMPK especially its prognostic role remains in controversy. Specifically, Zheng et al [14] reported that AMPK over-expression was correlated to a better prognosis among patients with hepatocellular carcinoma, while Baba et al [15] believed that the long-term survival of colorectal cancer was statistically irrelevant to AMPK expression levels. Since meta-analysis is a valuable tool to summarize conflicting literatures, we therefore performed this comprehensive meta-analysis in order to explain the prognostic significance of AMPK in human manlignancies and offer theoretical basis for future clinical applications.

## RESULTS

### General characteristics

**Figure 1:**
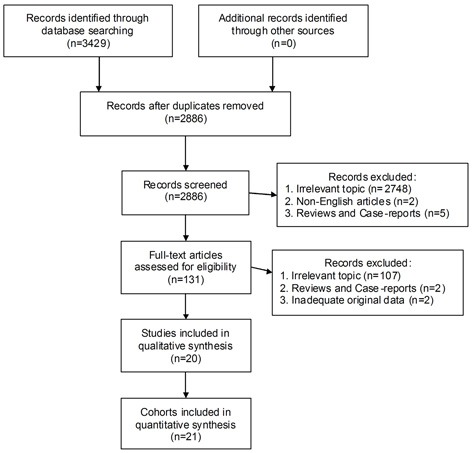
The selection flow chart of our pooled analysis

Among the initially retrieved 3429 entries, 20 studies were eventually included into the meta-analysis, which totally consist of 21 retrospective cohorts (Figure 1). The overall sample-size added up to 9987 participants, individually ranging from 42 to 3554. China was the chief source region of included investigations (n=7), followed by USA (n=6). Liver cancer (n=4), breast cancer (n=3) and gastric cancer (n=3) were the most frequent tumor types among included cohorts. The expression of AMPK was primarily detected by immunohistochemistry (n=14), in addition to microarray (n=3), polymerase chain reaction (n=2) and western blot (n=2). Data of mean-age and sex ratio were comparable among most cohorts, except for Kim 2013, Su 2014 and Zhang 2014-C2. More details of baseline characteristics were demonstrated in Table 1.

**Table 1:** Baseline characteristics of included studies

Study	Country	Tumor type	Detection method	p-AMPK or AMPK	TNM stage	AMPK level	Sample-size	Age	Sex (M/F)
Baba 2010 [15]	USA	Colorectal cancer	IHC	p-AMPK	I-IV	Low	309	NS	99/210
						High	409		160/249
Bhandaru 2014 [16]	Canada	Melanoma	IHC	AMPK	I-IV	Low	50	NS	30/20
						High	78		52/26
Buckendahl 2011 [17]	Germany	Ovarian cancer	IHC	AMPK	I-IV	Low	31	56.4±13.3	All female
						High	39		
Choi 2015 [18]	USA	Cervical cancer	IHC	AMPK	I-II	Low	51	42.1±12.0	All female
						High	71		
Fodor 2016 [19]	Hungary	Breast cancer	Microarray	AMPK	I-IV	Low	1792	NA	All female
						High	1762		
Guo 2015 [20]	USA	Lung cancer	IHC	p-AMPK	I-IV	Low	72	NA	NA
						High	122		
Hoffman 2013 [21]	USA	Lymphoma	Microarray	AMPK	I-IV	Low	456	61.9	All female
						High	527	62.3	
Kang 2012 [22]	Korea	Gastric cancer	IHC	p-AMPK	II-IV	Low	33	51.0±11.8	14/19
						High	40	60.0±8.0	25/15
Kim 2013 [23]	Korea	Gastric cancer	IHC	p-AMPK	I-IV	Low	242	61.0±15.3	140/102*
						High	379		258/121*
Lee 2012 [24]	China	Liver cancer	WB	AMPK	I-IV	Low	19	NA	14/5
						High	23		19/4
Li 2012 [25]	China	Ovarian cancer	PCR	AMPK	I-IV	Low	42	NS	All female
						High	34		
Su 2014 [26]	China	Squamous cell cancer of head and neck	IHC	p-AMPK	I-IV	Low	42	P=0.02*	39/3
						High	76		70/6
William 2012 [27]	USA	Lung cancer	Microarray	p-AMPK	I-IV	Low	147	65.0±12.0	77/70
						High	316	66.0±14.5	154/162
Xie 2015 [28]	USA	Glioma	PCR	AMPK	I-IV	Low	124	NA	NA
						High	230		
Zhang 2014-C1 [29]	UK	Breast cancer	IHC	AMPK	I-III	Low	80	56.0±9.8	All female
						High	83		
Zhang 2014-C2 [29]	UK	Breast cancer	IHC	AMPK	I-III	Low	162	**54.0**±13.5*	All female
						High	317		
Zhang 2015 [30]	China	Liver cancer	WB	p-AMPK	I-IV	Low	149	NS	121/105
						High	77		
Zheng 2013 [14]	China	Liver cancer	IHC	p-AMPK	I-III	Low	197	NS	165/32
						High	76		67/9
Zheng 2016 [31]	China	Liver cancer	IHC	p-AMPK	I-III	Low	145	NA	180/30
						High	65		
ZhengZ 2016 [32]	China	Gastric cancer	IHC	p-AMPK	I-IV	Low	628	NA	757/315
						High	444		
Zulato 2014 [33]	Italy	Colorectal cancer	IHC	p-AMPK	I-IV	Low	14	63.5±11.5	8/6
						High	34		21/13

M/F: male/female; IHC: immunohistochemistry; NS: not significant; NA: not available; WB: western blot; PCR: polymerase chain reaction; C1: cohort 1; C2: cohort 2;

* P<0.05.

### Methodological assessment

The majority of included trials were graded as high-quality in methodology, including three 8-score studies, thirteen 7-score studies and three 6-score studies. Only Kim 2013 and Zhang 2014-C2 were appraised as low-quality cohorts, each with 5 scores by Newcastle-Ottawa Scale (Table 2).

**Table 2:** Methodological assessment by Newcastle-Ottawa Scale

Study	Selection	Comparability	Outcome	Total
Baba 2010	3	1	3	7
Bhandaru 2014	3	2	2	7
Buckendahl 2011	3	2	3	8
Choi 2015	2	1	3	6
Fodor 2016	3	2	2	7
Guo 2015	3	2	2	7
Hoffman 2013	3	2	3	8
Kang 2012	2	2	3	7
Kim 2013	3	0	2	**5**
Lee 2012	3	2	2	7
Li 2012	3	2	2	7
Su 2014	3	1	3	7
William 2012	3	2	2	7
Xie 2015	3	2	2	7
Zhang 2014-C1	2	1	3	6
Zhang 2014-C2	2	1	2	**5**
Zhang 2015	3	2	2	7
Zheng 2013	2	1	3	6
Zheng 2016	2	2	3	7
ZhengZ 2016	3	1	3	7
Zulato 2014	3	2	3	8

### Prognostic significance of AMPK in survival analysis

**Figure 2:**
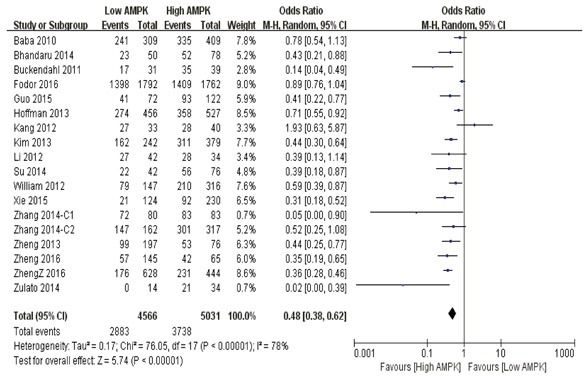
The correlation between AMPK expression levels and 3-year overall survival among cancer patients

**3-year overall survival** Compared with lower AMPK expression, patients featuring AMPK redundancy had significantly better outcome of 3-year overall survival (P<0.0001) (Figure 2).

**Figure 3:**
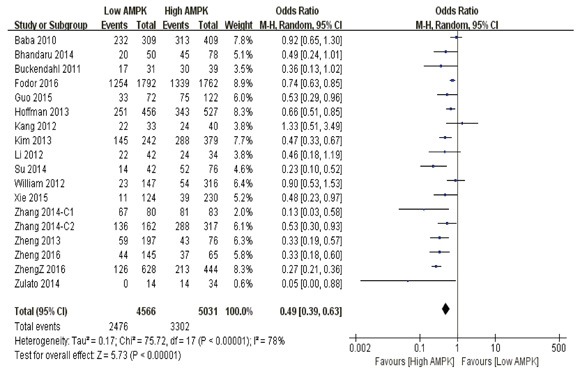
The correlation between AMPK expression levels and 5-year overall survival among cancer patients

**5-year overall survival** Our pooled analysis suggested that AMPK over-expression was a favorable indicator of 5-year overall survival among cancer suffers, in contrast to those with restricted levels (P<0.0001) (Figure 3).

**Figure 4:**
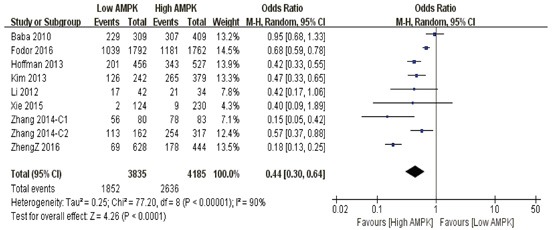
The correlation between AMPK expression levels and 10-year overall survival among cancer patients

**10-year overall survival** Patients with higher AMPK positivity were statistically correlated to better 10-year survival outcome than those with limited expression (P<0.0001) (Figure 4).

**3-year disease free survival** Patients with over-expressed AMPK had superior outcome of 3-year disease free survival compared to those with lower expression (P<0.0001) (Supplementary Figure S1).

**5-year disease free survival** Higher AMPK expression predicted favorable results of 5-year disease free survival within cancer participants, in comparison to those with lower expression (P=0.002) (Supplementary Figure S2).

**10-year disease free survival** In contrast to those with lower positivity, patients obtained better outcome of 10-year disease free survival in the presence of AMPK over-expression (P=0.0004) (Supplementary Figure S3).

### Subgroup analyses

#### Tumor type

**3-year overall survival** Higher AMPK level implicated a favorable 3-year overall survival among patients of gastric cancer (P=0.02), gynecological cancer (P=0.005), liver cancer (P<0.0001), lung cancer (P=0.0002) and other types (P=0.001). However, patients of breast cancer (P=0.20) and colorectal cancer (P=0.34) displayed similar prognosis regardless of different AMPK expressions (Supplementary Figure S4).

**5-year overall survival** AMPK over-expression was a beneficial indicator of 5-year overall survival amid participants of breast cancer (P=0.03), gastric cancer (P=0.02), gynecological cancer (P=0.01), liver cancer (P<0.0001) and other types (P=0.0008). Nevertheless, irrespective of colorectal cancer (P=0.41) and lung cancer (P=0.18), there was no significant difference between both compared groups (Supplementary Figure S5).

#### Sex ratio

**3-year overall survival** Irrespective of women-specific (P=0.006) or sex-unspecific study (P<0.0001), higher AMPK expression was associated with better 3-year overall survival among cancer patients (Supplementary Figure S6).

**5-year overall survival** In contrast to lower AMPK positivity, over-expression of AMPK suggested a superior outcome of 5-year overall survival among patients from both women-specific study (P<0.0001) and sex-unspecific study (P<0.0001) (Supplementary Figure S7).

#### Detection method

**3-year overall survival** Irrespective of immunohistochemistry (P<0.0001), microarray (P=0.02) and polymerase chain reaction (P<0.0001), the over-reactivity of AMPK was a potent predictor of better 3-year overall survival among cancer sufferers (Supplementary Figure S8).

**5-year overall survival** A better outcome of 5-year overall survival was observed among patients with higher level of AMPK expression, no matter it was detected by immunohistochemistry (P<0.0001), microarray (P<0.0001) or polymerase chain reaction (P=0.009) (Supplementary Figure S9).

#### AMPK activation status

**3-year overall survival** Higher expression of AMPK implied a favorable pooled outcome of 3-year overall survival among cancer patients, no matter in its normal (P=0.0002) or activated form (P<0.0001) (Supplementary Figure S10).

**5-year overall survival** Against lower expression level, a superior 5-year overall prognosis was obtained within patients featuring AMPK redundancy regardless of phosphorylated (P=0.0001) or original status (P<0.0001) (Supplementary Figure S11).

#### TNM stage

**3-year overall survival** Stronger AMPK positivity indicated longer 3-year overall survival among patients of I-IV (P<0.0001) and I-III (P<0.0001) stages. However, those with TNM II-IV stages had comparable survival outcome despite of different AMPK expression (P=0.25) (Supplementary Figure S12).

**5-year overall survival** Among participants with TNM I-IV (P<0.0001) and I-III (P<0.0001) stages, over-expression of AMPK was linked to better 5-year overall survival. Nevertheless, with regard to patients of II-IV stages, equivalent outcome of 5-year overall survival was obtained between lower and higher AMPK levels (P=0.56) (Supplementary Figure S13).

### Sensitivity analysis

Firstly, by excluding low-quality trials of Kim 2013 and Zhang 2014-C2, results of 3-year overall survival (P<0.0001), 5-year overall survival (P<0.0001), 10-year overall survival (P=0.0005), 3-year disease free survival (P<0.0001), 5-year disease free survival (P=0.03) and 10-year disease free survival (P=0.01) remained stable.

Secondly, by interchanging statistical modes between fixed-effects model and random-effects model, outcomes of 3-year overall survival (P<0.0001), 5-year overall survival (P<0.0001), 10-year overall survival (P<0.0001), 3-year disease free survival (P<0.0001), 5-year disease free survival (P<0.0001) and 10-year disease free survival (P=0.0004) remained unchanged.

Thirdly, by randomly removing included trials on STATA 12.0 platform, the outcome stability of 3-year overall survival (Supplementary Figure S14), 5-year overall survival (Supplementary Figure S15), 10-year overall survival (Supplementary Figure S16), 3-year disease free survival (Supplementary Figure S17), 5-year disease free survival (Supplementary Figure S18) and 10-year disease free survival (Supplementary Figure S19) was graphically confirmed.

### Publication bias

Take 3-year overall survival for example, both results of Begg's test (P=0.76) (Supplementary Figure S20) and Egger's test (P=0.09) (Supplementary Figure S21) verified that there was no publication bias within the included cohorts.

## DISCUSSION

According to our pooled results, higher expression of AMPK indicated better prognosis among cancer patients, irrespective of 3-year, 5-year, 10-year overall survival and disease free survival. This is the first conclusive evidence of the prognostic role of AMPK in human malignancies and the strength of the outcomes is quite persuasive since most of the P value is less than 0.00001. These conclusions seem reasonable and comprehensible based on the present consensus that as a major tumor suppressor, AMPK activation restricts the metastatic tendency and malignant dissemination of the primary lesion [34], which accounts for almost 90 percents of cancer relevant mortality. Moreover, phosphorylated AMPK could effectively inhibit cell growth and induce apoptosis within neoplastic tissues [35], jointly contributing to the favorable survival outcome among patients with higher AMPK expression. However, deviating from the majority of included cohorts, Kang et al [22] suggested that inhibition of AMPK could successfully induce gastric cancer cell apoptosis so that patients with lower expression level were observed to have better overall survival. Since all participants from Kang's study were followed up after a cisplatin-based adjuvant chemotherapy, whether this is simply an exception or cisplatin-based chemotherapy has unspecific correlations with AMPK expression awaits further clarifications.

In addition, subgroup analyses provided more in-depth perspectives of the AMPK significance. In terms of different tumor types, higher AMPK consistently served as an indicator of better prognosis among patients with gastric cancer, gynecological cancer, liver cancer and other kinds of cancer. On the other hand, its prognostic impact on the rest of malignancies remained ambiguous, especially for colorectal cancer. However, this outlier actually supports the experimental phenomenon by Baba et al [15] that AMPK signaling is only partially responsible for colorectal carcinogenesis and is activated merely in the setting of MAPK3/1 involvement. Since the positivity of MAPK3/1 is limited within colorectal cancer specimens, it is therefore explanatory that over-reactivity of AMPK is not a direct predictor of survival outcome among colorectal cancer patients. Besides, our pooled results confirmed that higher AMPK level revealed a better survival prognosis regardless of women-specific or sex-unspecific studies, ruling out the possibility of sex ratio as a confounding factor of internal heterogeneity. This pooled conclusion gives us a hint that AMPK may play a tumor suppressor role independent of estrogen and its downstream signaling among women-specific cancers, which calls for further mechanistic investigations. Moreover, irrespective of immunohistochemistry, microarray or polymerase chain reaction, higher AMPK expression was verified to positively correlate with survival expectancy of neoplastic sufferers. This result has confirmed the classical definition of AMPK that it bridges cellular molecules to construct an anti-tumor signaling network [36], since the upstream effectors could effectively trigger genomic transcription of AMPK before its downstream targets get stimulated. Thus more definite evidence is urgently required to explain the regulatory interplay between AMPK and the other essential factors. In terms of AMPK activation status, a favorable outcome of long-term survival was gained among patients with elevated AMPK expression, irrespective of phosphorylated or normal status. Since phosphorylated AMPK is actually the active form of AMPK, this result probably reveals that total AMPK expression is inductively increased along with the activating phosphorylation of AMPK, possibly mediated by self-stimulation of AMPK or its upstream kinase such as LKB1. What's more, the clinical TNM stage is commonly recognized as an interfering parameter of tumor prognosis. Nevertheless, we reported that the prognostic significance of AMPK over-expression was not distorted despite the disparity of TNM stages, which meant that AMPK may widely participate into various phases of tumorigenesis and cancer progression.

Besides the summary of pooled results, here we have an explanation of the usage of odds ratio as the effect-size model rather than hazard ratio. As we all known, survival analysis provides time-to-event data which hazard ratio fits best for. However, result of Cox regression is needed to transform Kaplan-Meier curves into hazard ratio, which is not available among most of current literatures. Furthermore, no matter how long the follow-up has lasted, there is merely one combined result deriving from the hazard ratio instead of the time-phased outcomes by odds ratio. Therefore we selected odds ratio as a statistical model as Ocana et al [37] and Badillo et al [38] recommended in Journal of the National Cancer Institute.

Although our meta-analysis was rigorously designed and performed, there were still some limitations. Firstly, all included cohorts were retrospectively analyzed, which might lead to poor internal comparability despite that the demographic characteristics were statistically comparable. Prospective and randomized trials are therefore needed to draw a more persuasive conclusion in future updates. Secondly, although the total sample-size nearly reached 10 thousands, the amount of included studies were still insufficient, especially for the subgroup analyses. Thirdly, despite that we had conducted enough subgroup analyses, the internal heterogeneity could not be fully eliminated, which hinted the existence of unclear confounding elements.

Taken together, this is the first meta-analysis which confirms that higher expression of AMPK is correlated to better prognosis of 3-year, 5-year, 10-year overall and disease free survival among cancer sufferers. Additionally, these prognostic tendencies are independent of tumor type, sex ratio, detection method, AMPK activation status and TNM stage. Therefore, we believe that AMPK targeted therapy is a promising and revolutionary strategy for cancer patients.

## MATERIALS AND METHODS

All procedures mentioned below were performed in accord with PRISMA Checklist and Cochrane Collaboration protocols. Two investigators carried out each step independently, while any discrepancy was resolved by mutual discussion.

### Literature search

Databases of PubMed, Web of Science, Embase and Cochrane Library were thoroughly examined using the search term “ampk AND (cancer OR carcinoma OR malignancy OR tumor)”. Both abstract and full-text of the preliminary entries were screened in order to guarantee the eligibility of included studies.

### Selection criteria

Studies that met the following criteria were eventually included: 1. Formally published and English-written articles until May 2016; 2. Studies that comparing the prognostic value of different AMPK expression in human malignancies;

Studies were eliminated due to the following reasons: 1. Overlapped or duplicated articles; 2. Inappropriate article type including reviews and case-reports; 3. Inadequate original data of survival analysis;

### Methodological assessment

Since all of the eligible studies were observational cohorts, the Newcastle-Ottawa Scale was therefore utilized for methodological appraisal. There were totally three categories within the scale including selection, comparability and outcome, with a full-mark of nine. Studies were identified as high-quality in methodology with at least six scores.

### Data extraction

A standardized form was designed for purpose of data extraction. Details of baseline characteristics (country; tumor type; detection method; AMPK activation status; TNM stage; groups; sample size; age; sex) were extracted from the main text or tables among the included documents. Survival data (3-year overall survival; 5-year overall survival; 10-year overall survival; 3-year disease free survival; 5-year disease free survival; 10-year disease free survival;) was mainly obtained from Kaplan-Meier curves, with graphical assistance by Engauge Digitizer 4.1.

### Statistical analysis

Review Manager 5.3 was employed as a statistical platform for pooled analysis. The effect size of each endpoint was presented by odds ratio and 95% confidence interval. I^2^ signified the degree of inconsistency across the included studies, whose value <25%, <50% and >50% implied low, moderate and severe heterogeneity respectively. Fixed-effects model was best-fit under circumstance of low heterogeneity, while random-effects model was the optimal choice for the remaining situations. Subgroup analyses were additionally conducted to seek for the potential confounding factors (tumor type; sex; detection method; AMPK activation status; TNM stage) within. By excluding low-quality trials and interchanging statistical modes, the stability of pooled outcomes was tested by sensitivity analysis. Moreover, Egger's test and Begg's test were applied to inspect publication bias among included trials. P<0.05 denoted statistical significance between the comparison.

## SUPPLEMENTARY FIGURES


