# Supplementary material for: Prognostic significance of AMPK in human malignancies: A meta-analysis
Source: Oncotarget. 2016 Oct 3;7(46):75739–48. doi: 10.18632/oncotarget.12405 (PMC5342774; doi:10.18632/oncotarget.12405)
Supplement: Supplementary file 1 [file oncotarget-07-75739-s001.pdf]

Prognostic significance of AMPK in human malignancies: A meta-analysis

SUPPLEMENTARY FIGURES

Study or Subgroup	Low AMPK Events	Low AMPK Total	High AMPK Events	High AMPK Total	Weight	Odds Ratio M-H, Random, 95% CI
Choi 2015	44	51	64	71	4.0%	0.69 [0.23, 2.10]
Kang 2012	21	33	20	40	5.3%	1.75 [0.68, 4.49]
Kim 2013	153	242	296	379	17.6%	0.48 [0.34, 0.69]
Lee 2012	6	19	14	23	3.2%	0.30 [0.08, 1.07]
Li 2012	23	42	25	34	5.0%	0.44 [0.16, 1.15]
William 2012	55	147	170	316	16.0%	0.51 [0.34, 0.77]
Zhang 2014-C1	68	80	79	83	3.6%	0.29 [0.09, 0.93]
Zhang 2014-C2	131	162	276	317	12.5%	0.63 [0.38, 1.05]
Zhang 2015	56	149	35	77	11.3%	0.72 [0.41, 1.26]
Zheng 2013	57	197	42	76	11.6%	0.33 [0.19, 0.57]
Zheng 2016	36	145	33	65	10.0%	0.32 [0.17, 0.59]
Zulato 2014	0	14	0	34		Not estimable
**Total (95% CI)**		**1281**		**1515**	**100.0%**	**0.51 [0.40, 0.64]**
Total events	650		1054			

Heterogeneity: Tau² = 0.05; Chi² = 15.41, df = 10 (P = 0.12); I² = 35%
Test for overall effect: Z = 5.55 (P < 0.00001)

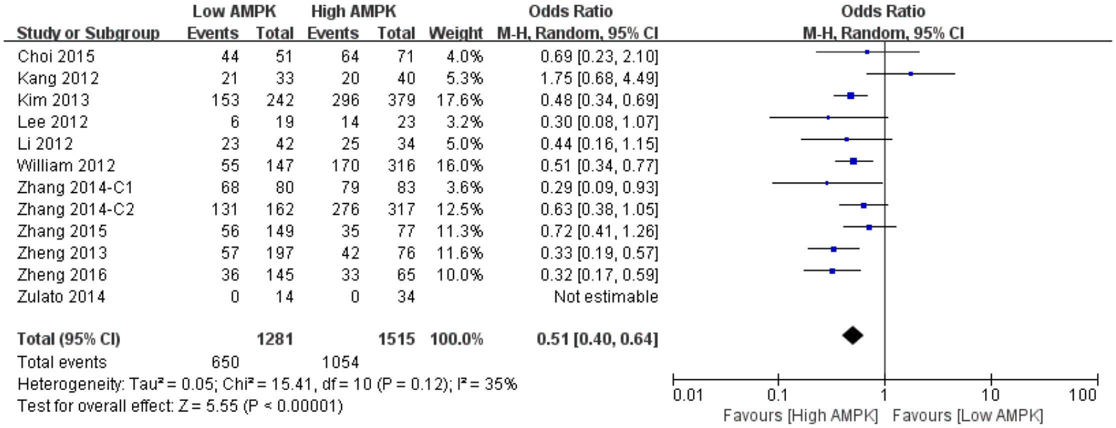

Supplementary Figure S1: The correlation between AMPK expression levels and 3-year disease free survival among cancer patients.

Study or Subgroup	Low AMPK Events	Low AMPK Total	High AMPK Events	High AMPK Total	Weight	Odds Ratio M-H, Random, 95% CI
Choi 2015	44	51	61	71	7.8%	1.03 [0.36, 2.92]
Kang 2012	19	33	9	40	8.0%	4.67 [1.70, 12.88]
Kim 2013	145	242	285	379	12.2%	0.49 [0.35, 0.70]
Lee 2012	0	19	12	23	2.1%	0.02 [0.00, 0.44]
Li 2012	19	42	25	34	8.2%	0.30 [0.11, 0.79]
William 2012	13	147	38	316	10.3%	0.71 [0.37, 1.38]
Zhang 2014-C1	62	80	77	83	8.2%	0.27 [0.10, 0.72]
Zhang 2014-C2	122	162	266	317	11.5%	0.58 [0.37, 0.93]
Zhang 2015	36	149	24	77	10.6%	0.70 [0.38, 1.30]
Zheng 2013	27	197	36	76	10.7%	0.18 [0.10, 0.32]
Zheng 2016	25	145	31	65	10.4%	0.23 [0.12, 0.44]
Zulato 2014	0	14	0	34		Not estimable
**Total (95% CI)**		**1281**		**1515**	**100.0%**	**0.49 [0.31, 0.77]**
Total events	512		864			

Heterogeneity: Tau² = 0.42; Chi² = 46.83, df = 10 (P < 0.00001); I² = 79%

Test for overall effect: Z = 3.06 (P = 0.002)

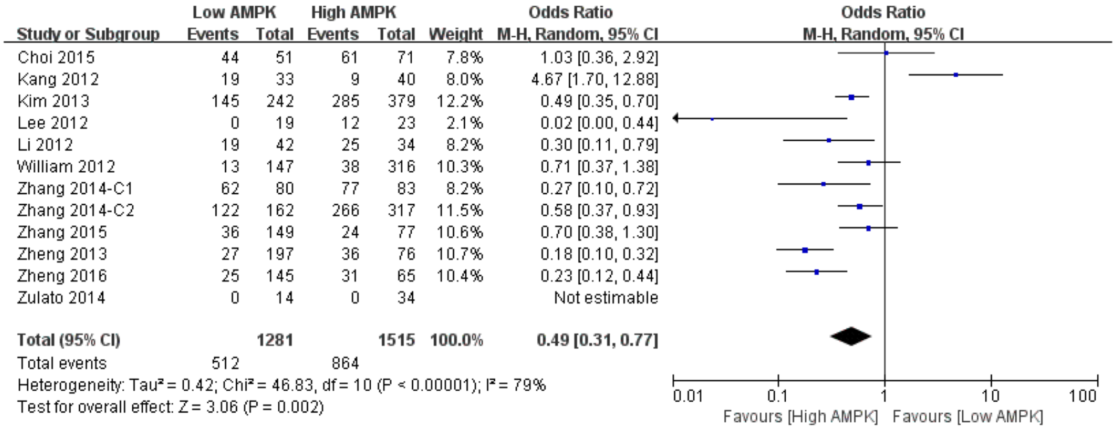

Supplementary Figure S2: The correlation between AMPK expression levels and 5-year disease free survival among cancer patients.

Study or Subgroup	Low AMPK Events	Low AMPK Total	High AMPK Events	High AMPK Total	Weight	Odds Ratio M-H, Random, 95% CI
Choi 2015	44	51	61	71	11.0%	1.03 [0.36, 2.92]
Kim 2013	126	242	265	379	29.5%	0.47 [0.33, 0.65]
Li 2012	12	42	21	34	12.2%	0.25 [0.09, 0.65]
Zhang 2014-C1	55	80	76	83	13.2%	0.20 [0.08, 0.50]
Zhang 2014-C2	101	162	222	317	27.3%	0.71 [0.48, 1.06]
Zhang 2015	4	149	4	77	6.9%	0.50 [0.12, 2.07]
**Total (95% CI)**		**726**		**961**	**100.0%**	**0.48 [0.31, 0.72]**
Total events	342		649			

Heterogeneity: Tau² = 0.12; Chi² = 10.78, df = 5 (P = 0.06); I² = 54%

Test for overall effect: Z = 3.53 (P = 0.0004)

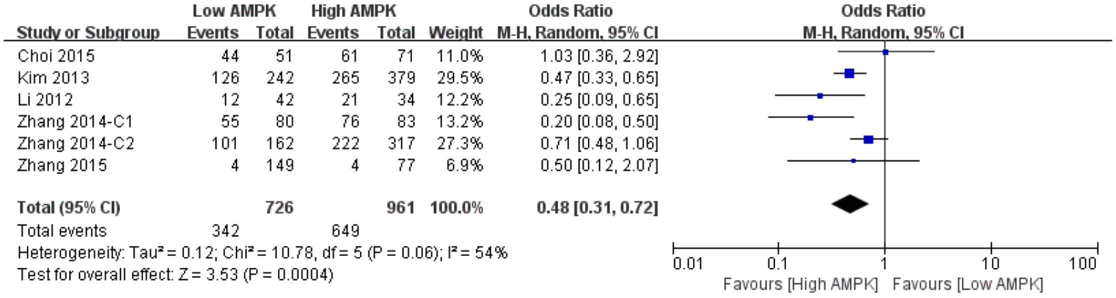

Supplementary Figure S3: The correlation between AMPK expression levels and 10-year disease free survival among cancer patients.

Study or Subgroup	Low AMPK Events	Low AMPK Total	High AMPK Events	High AMPK Total	Weight	Odds Ratio M-H, Random, 95% CI
**3.1.1 Breast cancer**						
Fodor 2016	1398	1792	1409	1762	9.0%	0.89 [0.76, 1.04]
Zhang 2014-C1	72	80	83	83	0.7%	0.05 [0.00, 0.90]
Zhang 2014-C2	147	162	301	317	5.2%	0.52 [0.25, 1.08]
**Subtotal (95% CI)**		**2034**		**2162**	**14.9%**	**0.61 [0.29, 1.30]**
Total events	1617		1793			
Heterogeneity: Tau² = 0.26; Chi² = 5.72, df = 2 (P = 0.06); I² = 65%						
Test for overall effect: Z = 1.28 (P = 0.20)						
**3.1.2 Gastric cancer**						
Kang 2012	27	33	28	40	3.3%	1.93 [0.63, 5.87]
Kim 2013	162	242	311	379	7.7%	0.44 [0.30, 0.64]
ZhengZ 2016	176	628	231	444	8.6%	0.36 [0.28, 0.46]
**Subtotal (95% CI)**		**903**		**863**	**19.6%**	**0.51 [0.30, 0.88]**
Total events	365		570			
Heterogeneity: Tau² = 0.16; Chi² = 8.63, df = 2 (P = 0.01); I² = 77%						
Test for overall effect: Z = 2.42 (P = 0.02)						
**3.1.3 Colorectal cancer**						
Baba 2010	241	309	335	409	7.8%	0.78 [0.54, 1.13]
Zulato 2014	0	14	21	34	0.7%	0.02 [0.00, 0.39]
**Subtotal (95% CI)**		**323**		**443**	**8.5%**	**0.17 [0.00, 6.24]**
Total events	241		356			
Heterogeneity: Tau² = 5.76; Chi² = 6.18, df = 1 (P = 0.01); I² = 84%						
Test for overall effect: Z = 0.96 (P = 0.34)						
**3.1.4 Gynecological cancer**						
Buckendahl 2011	17	31	35	39	2.8%	0.14 [0.04, 0.49]
Li 2012	27	42	28	34	3.4%	0.39 [0.13, 1.14]
**Subtotal (95% CI)**		**73**		**73**	**6.2%**	**0.24 [0.09, 0.66]**
Total events	44		63			
Heterogeneity: Tau² = 0.17; Chi² = 1.46, df = 1 (P = 0.23); I² = 32%						
Test for overall effect: Z = 2.78 (P = 0.005)						
**3.1.5 Liver cancer**						
Zheng 2013	99	197	53	76	6.3%	0.44 [0.25, 0.77]
Zheng 2016	57	145	42	65	6.0%	0.35 [0.19, 0.65]
**Subtotal (95% CI)**		**342**		**141**	**12.4%**	**0.40 [0.26, 0.60]**
Total events	156		95			
Heterogeneity: Tau² = 0.00; Chi² = 0.25, df = 1 (P = 0.62); I² = 0%						
Test for overall effect: Z = 4.38 (P < 0.0001)						
**3.1.6 Lung cancer**						
Guo 2015	41	72	93	122	5.9%	0.41 [0.22, 0.77]
William 2012	79	147	210	316	7.6%	0.59 [0.39, 0.87]
**Subtotal (95% CI)**		**219**		**438**	**13.5%**	**0.53 [0.38, 0.74]**
Total events	120		303			
Heterogeneity: Tau² = 0.00; Chi² = 0.86, df = 1 (P = 0.35); I² = 0%						
Test for overall effect: Z = 3.70 (P = 0.0002)						
**3.1.7 Others**						
Bhandaru 2014	23	50	52	78	5.2%	0.43 [0.21, 0.88]
Hoffman 2013	274	456	358	527	8.5%	0.71 [0.55, 0.92]
Su 2014	22	42	56	76	4.8%	0.39 [0.18, 0.87]
Xie 2015	21	124	92	230	6.5%	0.31 [0.18, 0.52]
**Subtotal (95% CI)**		**672**		**911**	**25.1%**	**0.46 [0.29, 0.74]**
Total events	340		558			
Heterogeneity: Tau² = 0.15; Chi² = 9.34, df = 3 (P = 0.03); I² = 68%						
Test for overall effect: Z = 3.19 (P = 0.001)						
**Total (95% CI)**		**4566**		**5031**	**100.0%**	**0.48 [0.38, 0.62]**
Total events	2883		3738			
Heterogeneity: Tau² = 0.17; Chi² = 76.05, df = 17 (P < 0.00001); I² = 78%						
Test for overall effect: Z = 5.74 (P < 0.00001)						
Test for subgroup differences: Chi² = 3.65, df = 6 (P = 0.72), I² = 0%						

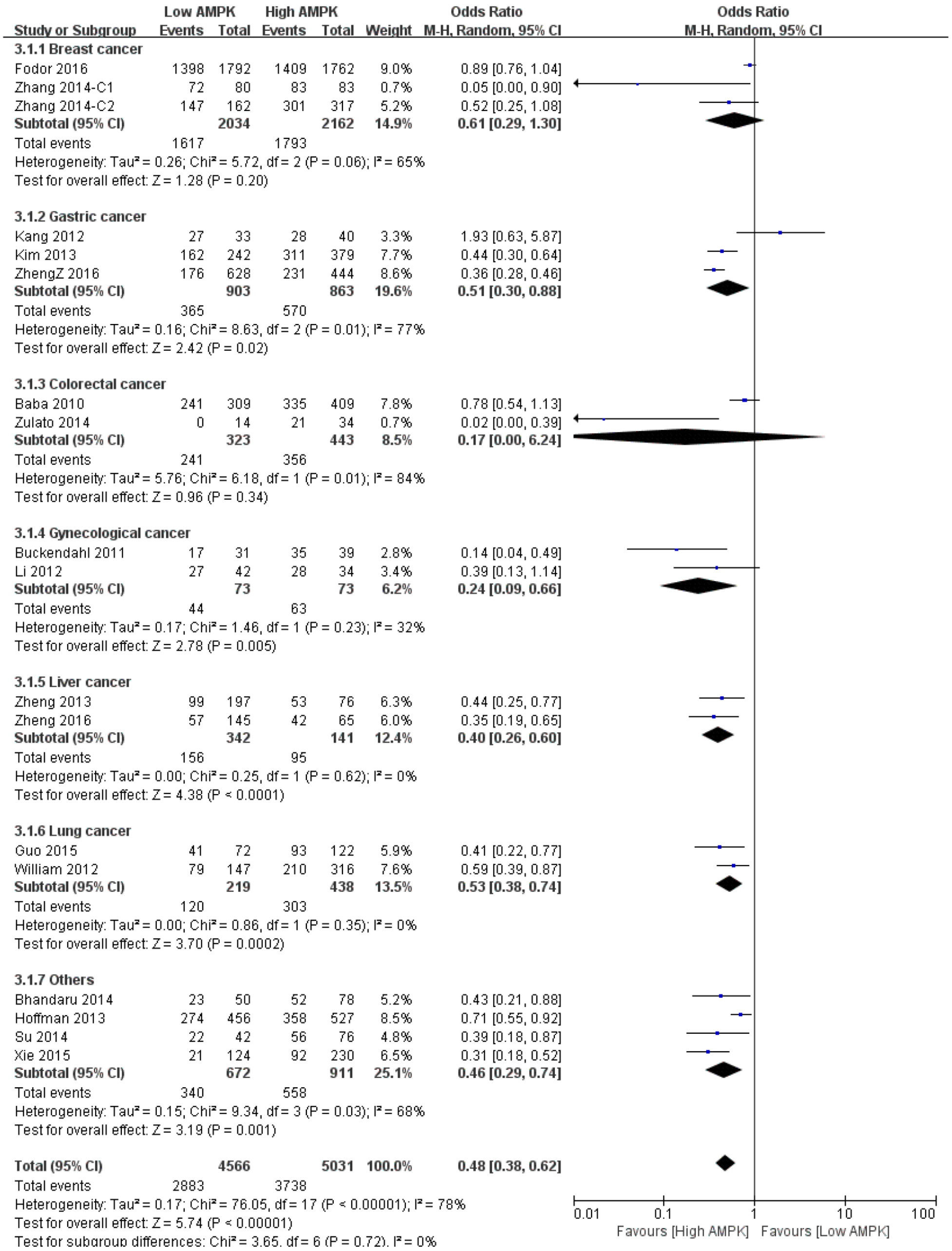

Supplementary Figure S4: The correlation between AMPK expression levels and 3-year overall survival among patients with different tumor types.

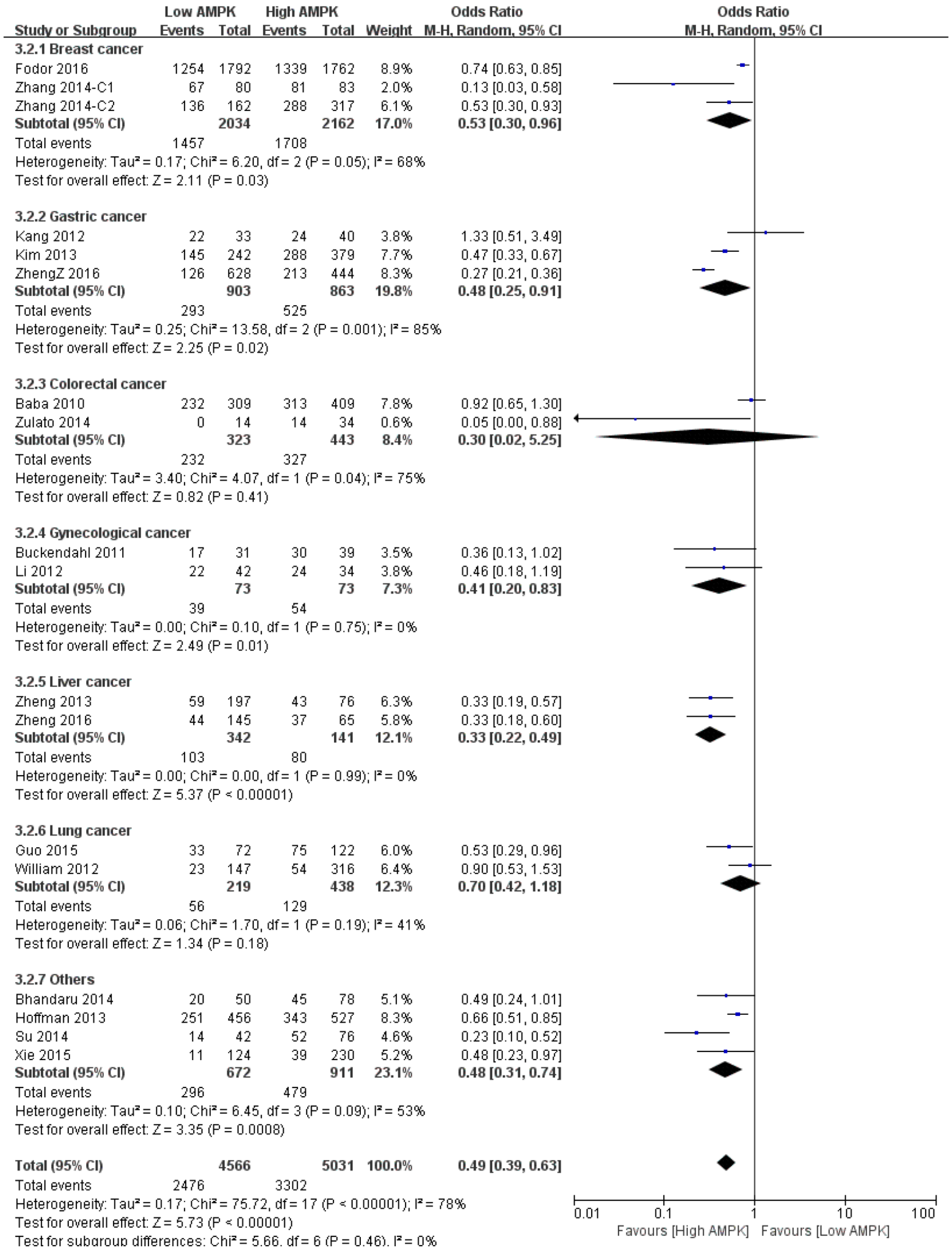

Supplementary Figure S5: The correlation between AMPK expression levels and 5-year overall survival among patients with different tumor types.

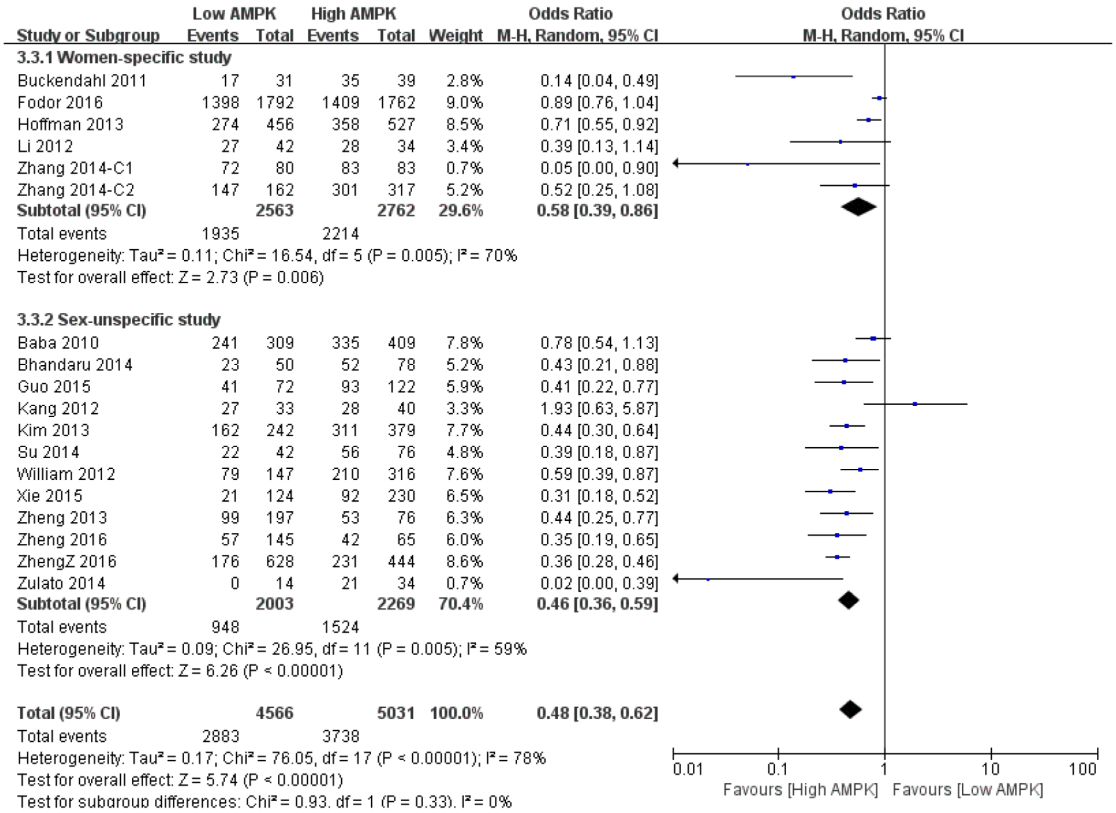

Supplementary Figure S6: The correlation between AMPK expression levels and 3-year overall survival among patients with sex ratio.

Study or Subgroup	Low AMPK Events	Low AMPK Total	High AMPK Events	High AMPK Total	Weight	Odds Ratio M-H, Random, 95% CI
**3.4.1 Women-specific study**						
Buckendahl 2011	17	31	30	39	3.5%	0.36 [0.13, 1.02]
Fodor 2016	1254	1792	1339	1762	8.9%	0.74 [0.63, 0.85]
Hoffman 2013	251	456	343	527	8.3%	0.66 [0.51, 0.85]
Li 2012	22	42	24	34	3.8%	0.46 [0.18, 1.19]
Zhang 2014-C1	67	80	81	83	2.0%	0.13 [0.03, 0.58]
Zhang 2014-C2	136	162	288	317	6.1%	0.53 [0.30, 0.93]
**Subtotal (95% CI)**		**2563**		**2762**	**32.6%**	**0.61 [0.48, 0.78]**
Total events	1747		2105			
Heterogeneity: Tau² = 0.03; Chi² = 8.66, df = 5 (P = 0.12); I² = 42%						
Test for overall effect: Z = 3.98 (P < 0.0001)						
**3.4.2 Sex-unspecific study**						
Baba 2010	232	309	313	409	7.8%	0.92 [0.65, 1.30]
Bhandaru 2014	20	50	45	78	5.1%	0.49 [0.24, 1.01]
Guo 2015	33	72	75	122	6.0%	0.53 [0.29, 0.96]
Kang 2012	22	33	24	40	3.8%	1.33 [0.51, 3.49]
Kim 2013	145	242	288	379	7.7%	0.47 [0.33, 0.67]
Su 2014	14	42	52	76	4.6%	0.23 [0.10, 0.52]
William 2012	23	147	54	316	6.4%	0.90 [0.53, 1.53]
Xie 2015	11	124	39	230	5.2%	0.48 [0.23, 0.97]
Zheng 2013	59	197	43	76	6.3%	0.33 [0.19, 0.57]
Zheng 2016	44	145	37	65	5.8%	0.33 [0.18, 0.60]
ZhengZ 2016	126	628	213	444	8.3%	0.27 [0.21, 0.36]
Zulato 2014	0	14	14	34	0.6%	0.05 [0.00, 0.88]
**Subtotal (95% CI)**		**2003**		**2269**	**67.4%**	**0.47 [0.34, 0.66]**
Total events	729		1197			
Heterogeneity: Tau² = 0.24; Chi² = 49.11, df = 11 (P < 0.00001); I² = 78%						
Test for overall effect: Z = 4.37 (P < 0.0001)						
**Total (95% CI)**		**4566**		**5031**	**100.0%**	**0.49 [0.39, 0.63]**
Total events	2476		3302			
Heterogeneity: Tau² = 0.17; Chi² = 75.72, df = 17 (P < 0.00001); I² = 78%						
Test for overall effect: Z = 5.73 (P < 0.00001)						
Test for subgroup differences: Chi² = 1.47, df = 1 (P = 0.23), I² = 32.0%						

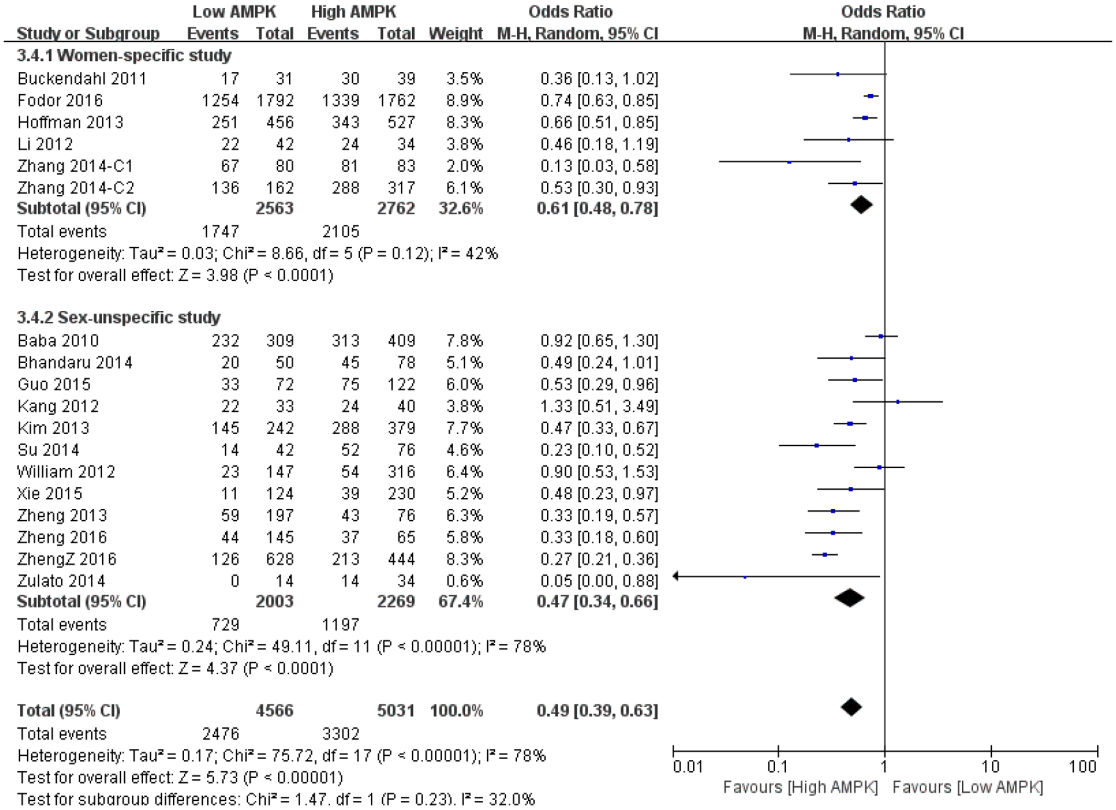

Supplementary Figure S7: The correlation between AMPK expression levels and 5-year overall survival among patients with different sex ratio.

Study or Subgroup	Low AMPK Events	Low AMPK Total	High AMPK Events	High AMPK Total	Weight	Odds Ratio M-H, Random, 95% CI
**3.5.1 IHC**						
Baba 2010	241	309	335	409	7.8%	0.78 [0.54, 1.13]
Bhandaru 2014	23	50	52	78	5.2%	0.43 [0.21, 0.88]
Buckendahl 2011	17	31	35	39	2.8%	0.14 [0.04, 0.49]
Guo 2015	41	72	93	122	5.9%	0.41 [0.22, 0.77]
Kang 2012	27	33	28	40	3.3%	1.93 [0.63, 5.87]
Kim 2013	162	242	311	379	7.7%	0.44 [0.30, 0.64]
Su 2014	22	42	56	76	4.8%	0.39 [0.18, 0.87]
Zhang 2014-C1	72	80	83	83	0.7%	0.05 [0.00, 0.90]
Zhang 2014-C2	147	162	301	317	5.2%	0.52 [0.25, 1.08]
Zheng 2013	99	197	53	76	6.3%	0.44 [0.25, 0.77]
Zheng 2016	57	145	42	65	6.0%	0.35 [0.19, 0.65]
ZhengZ 2016	176	628	231	444	8.6%	0.36 [0.28, 0.46]
Zulato 2014	0	14	21	34	0.7%	0.02 [0.00, 0.39]
**Subtotal (95% CI)**		**2005**		**2162**	**65.0%**	**0.44 [0.33, 0.58]**
Total events	1084		1641			
Heterogeneity: Tau² = 0.12; Chi² = 29.05, df = 12 (P = 0.004); I² = 59%						
Test for overall effect: Z = 5.84 (P < 0.00001)						
**3.5.2 Microarray**						
Fodor 2016	1398	1792	1409	1762	9.0%	0.89 [0.76, 1.04]
Hoffman 2013	274	456	358	527	8.5%	0.71 [0.55, 0.92]
William 2012	79	147	210	316	7.6%	0.59 [0.39, 0.87]
**Subtotal (95% CI)**		**2395**		**2605**	**25.1%**	**0.76 [0.60, 0.95]**
Total events	1751		1977			
Heterogeneity: Tau² = 0.02; Chi² = 4.73, df = 2 (P = 0.09); I² = 58%						
Test for overall effect: Z = 2.39 (P = 0.02)						
**3.5.4 PCR**						
Li 2012	27	42	28	34	3.4%	0.39 [0.13, 1.14]
Xie 2015	21	124	92	230	6.5%	0.31 [0.18, 0.52]
**Subtotal (95% CI)**		**166**		**264**	**9.9%**	**0.32 [0.20, 0.52]**
Total events	48		120			
Heterogeneity: Tau² = 0.00; Chi² = 0.14, df = 1 (P = 0.71); I² = 0%						
Test for overall effect: Z = 4.63 (P < 0.00001)						
**Total (95% CI)**		**4566**		**5031**	**100.0%**	**0.48 [0.38, 0.62]**
Total events	2883		3738			
Heterogeneity: Tau² = 0.17; Chi² = 76.05, df = 17 (P < 0.00001); I² = 78%						
Test for overall effect: Z = 5.74 (P < 0.00001)						
Test for subgroup differences: Chi² = 14.58, df = 2 (P = 0.0007), I² = 86.3%						

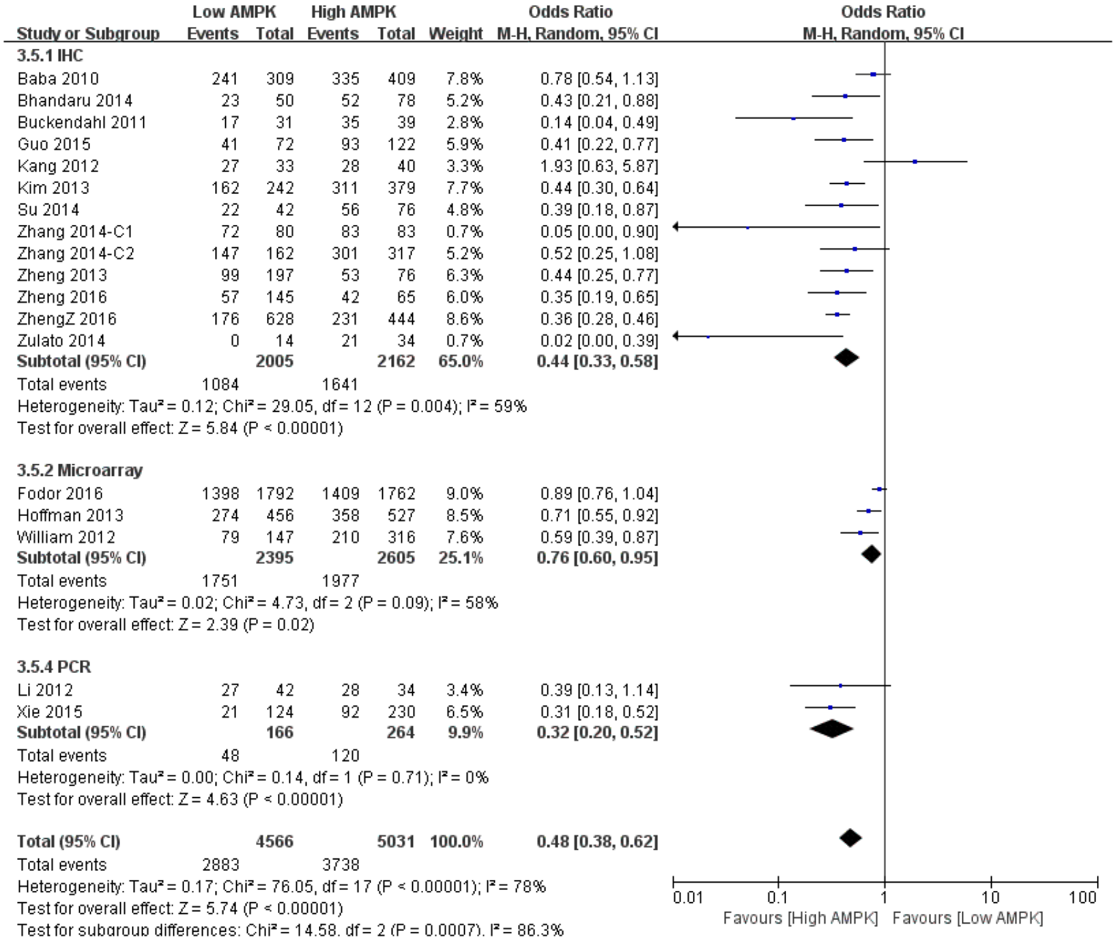

Supplementary Figure S8: The correlation between AMPK expression levels and 3-year overall survival among patients with detection methods.

Study or Subgroup	Low AMPK Events	Low AMPK Total	High AMPK Events	High AMPK Total	Weight	Odds Ratio M-H, Random, 95% CI
**3.6.1 IHC**						
Baba 2010	232	309	313	409	7.8%	0.92 [0.65, 1.30]
Bhandaru 2014	20	50	45	78	5.1%	0.49 [0.24, 1.01]
Buckendahl 2011	17	31	30	39	3.5%	0.36 [0.13, 1.02]
Guo 2015	33	72	75	122	6.0%	0.53 [0.29, 0.96]
Kang 2012	22	33	24	40	3.8%	1.33 [0.51, 3.49]
Kim 2013	145	242	288	379	7.7%	0.47 [0.33, 0.67]
Su 2014	14	42	52	76	4.6%	0.23 [0.10, 0.52]
Zhang 2014-C1	67	80	81	83	2.0%	0.13 [0.03, 0.58]
Zhang 2014-C2	136	162	288	317	6.1%	0.53 [0.30, 0.93]
Zheng 2013	59	197	43	76	6.3%	0.33 [0.19, 0.57]
Zheng 2016	44	145	37	65	5.8%	0.33 [0.18, 0.60]
ZhengZ 2016	126	628	213	444	8.3%	0.27 [0.21, 0.36]
Zulato 2014	0	14	14	34	0.6%	0.05 [0.00, 0.88]
**Subtotal (95% CI)**		**2005**		**2162**	**67.4%**	**0.43 [0.31, 0.59]**
Total events	915		1503			
Heterogeneity: Tau² = 0.21; Chi² = 45.39, df = 12 (P < 0.00001); I² = 74%						
Test for overall effect: Z = 5.21 (P < 0.00001)						
**3.6.2 Microarray**						
Fodor 2016	1254	1792	1339	1762	8.9%	0.74 [0.63, 0.85]
Hoffman 2013	251	456	343	527	8.3%	0.66 [0.51, 0.85]
William 2012	23	147	54	316	6.4%	0.90 [0.53, 1.53]
**Subtotal (95% CI)**		**2395**		**2605**	**23.6%**	**0.72 [0.64, 0.82]**
Total events	1528		1736			
Heterogeneity: Tau² = 0.00; Chi² = 1.24, df = 2 (P = 0.54); I² = 0%						
Test for overall effect: Z = 5.04 (P < 0.00001)						
**3.6.4 PCR**						
Li 2012	22	42	24	34	3.8%	0.46 [0.18, 1.19]
Xie 2015	11	124	39	230	5.2%	0.48 [0.23, 0.97]
**Subtotal (95% CI)**		**166**		**264**	**8.9%**	**0.47 [0.27, 0.83]**
Total events	33		63			
Heterogeneity: Tau² = 0.00; Chi² = 0.00, df = 1 (P = 0.95); I² = 0%						
Test for overall effect: Z = 2.60 (P = 0.009)						
**Total (95% CI)**		**4566**		**5031**	**100.0%**	**0.49 [0.39, 0.63]**
Total events	2476		3302			
Heterogeneity: Tau² = 0.17; Chi² = 75.72, df = 17 (P < 0.00001); I² = 78%						
Test for overall effect: Z = 5.73 (P < 0.00001)						
Test for subgroup differences: Chi² = 10.64, df = 2 (P = 0.005), I² = 81.2%						

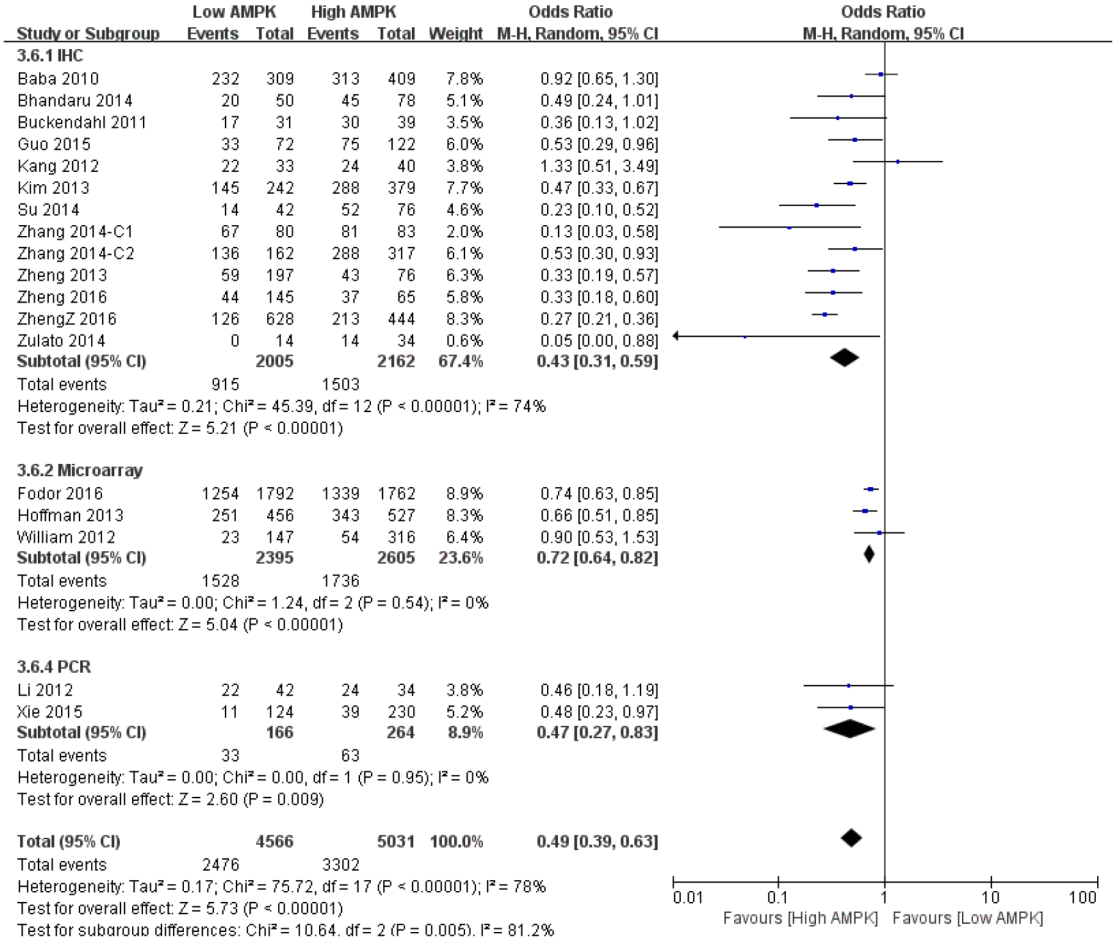

Supplementary Figure S9: The correlation between AMPK expression levels and 5-year overall survival among patients with detection methods.

Study or Subgroup	Low AMPK Events	Low AMPK Total	High AMPK Events	High AMPK Total	Weight	Odds Ratio M-H, Random, 95% CI
**3.7.1 p-AMPK**						
Baba 2010	241	309	335	409	7.8%	0.78 [0.54, 1.13]
Guo 2015	41	72	93	122	5.9%	0.41 [0.22, 0.77]
Kang 2012	27	33	28	40	3.3%	1.93 [0.63, 5.87]
Kim 2013	162	242	311	379	7.7%	0.44 [0.30, 0.64]
Su 2014	22	42	56	76	4.8%	0.39 [0.18, 0.87]
William 2012	79	147	210	316	7.6%	0.59 [0.39, 0.87]
Zheng 2013	99	197	53	76	6.3%	0.44 [0.25, 0.77]
Zheng 2016	57	145	42	65	6.0%	0.35 [0.19, 0.65]
ZhengZ 2016	176	628	231	444	8.6%	0.36 [0.28, 0.46]
Zulato 2014	0	14	21	34	0.7%	0.02 [0.00, 0.39]
**Subtotal (95% CI)**		**1829**		**1961**	**58.7%**	**0.48 [0.37, 0.64]**
Total events	904		1380			
Heterogeneity: Tau² = 0.11; Chi² = 24.69, df = 9 (P = 0.003); I² = 64%						
Test for overall effect: Z = 5.16 (P < 0.00001)						
**3.7.2 AMPK**						
Bhandaru 2014	23	50	52	78	5.2%	0.43 [0.21, 0.88]
Buckendahl 2011	17	31	35	39	2.8%	0.14 [0.04, 0.49]
Fodor 2016	1398	1792	1409	1762	9.0%	0.89 [0.76, 1.04]
Hoffman 2013	274	456	358	527	8.5%	0.71 [0.55, 0.92]
Li 2012	27	42	28	34	3.4%	0.39 [0.13, 1.14]
Xie 2015	21	124	92	230	6.5%	0.31 [0.18, 0.52]
Zhang 2014-C1	72	80	83	83	0.7%	0.05 [0.00, 0.90]
Zhang 2014-C2	147	162	301	317	5.2%	0.52 [0.25, 1.08]
**Subtotal (95% CI)**		**2737**		**3070**	**41.3%**	**0.48 [0.32, 0.71]**
Total events	1979		2358			
Heterogeneity: Tau² = 0.18; Chi² = 30.14, df = 7 (P < 0.0001); I² = 77%						
Test for overall effect: Z = 3.69 (P = 0.0002)						
**Total (95% CI)**		**4566**		**5031**	**100.0%**	**0.48 [0.38, 0.62]**
Total events	2883		3738			
Heterogeneity: Tau² = 0.17; Chi² = 76.05, df = 17 (P < 0.00001); I² = 78%						
Test for overall effect: Z = 5.74 (P < 0.00001)						
Test for subgroup differences: Chi² = 0.00, df = 1 (P = 0.97), I² = 0%						

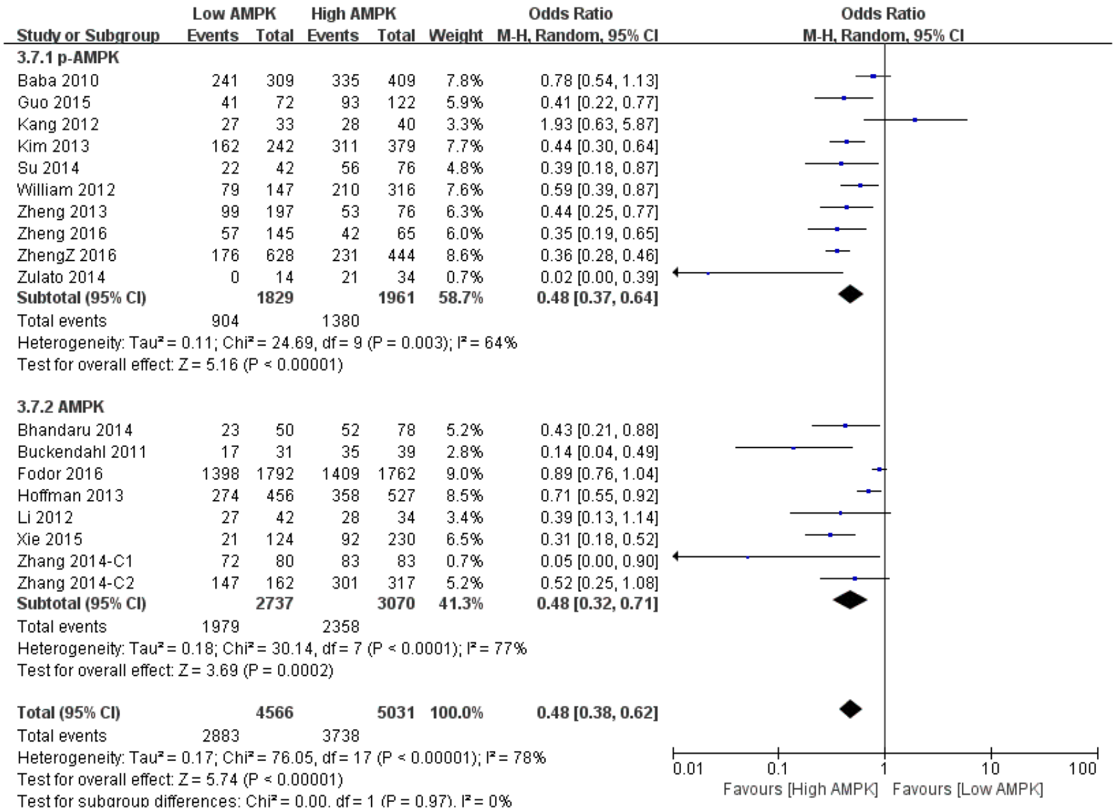

Supplementary Figure S10: The correlation between AMPK expression levels and 3-year overall survival among patients with different AMPK activation status.

Study or Subgroup	Low AMPK Events	Low AMPK Total	High AMPK Events	High AMPK Total	Weight	Odds Ratio M-H, Random, 95% CI
**3.8.1 p-AMPK**						
Baba 2010	232	309	313	409	7.8%	0.92 [0.65, 1.30]
Guo 2015	33	72	75	122	6.0%	0.53 [0.29, 0.96]
Kang 2012	22	33	24	40	3.8%	1.33 [0.51, 3.49]
Kim 2013	145	242	288	379	7.7%	0.47 [0.33, 0.67]
Su 2014	14	42	52	76	4.6%	0.23 [0.10, 0.52]
William 2012	23	147	54	316	6.4%	0.90 [0.53, 1.53]
Zheng 2013	59	197	43	76	6.3%	0.33 [0.19, 0.57]
Zheng 2016	44	145	37	65	5.8%	0.33 [0.18, 0.60]
ZhengZ 2016	126	628	213	444	8.3%	0.27 [0.21, 0.36]
Zulato 2014	0	14	14	34	0.6%	0.05 [0.00, 0.88]
**Subtotal (95% CI)**		**1829**		**1961**	**57.2%**	**0.47 [0.32, 0.70]**
Total events	698		1113			
Heterogeneity: Tau² = 0.28; Chi² = 49.06, df = 9 (P < 0.00001); I² = 82%						
Test for overall effect: Z = 3.79 (P = 0.0001)						
**3.8.2 AMPK**						
Bhandaru 2014	20	50	45	78	5.1%	0.49 [0.24, 1.01]
Buckendahl 2011	17	31	30	39	3.5%	0.36 [0.13, 1.02]
Fodor 2016	1254	1792	1339	1762	8.9%	0.74 [0.63, 0.85]
Hoffman 2013	251	456	343	527	8.3%	0.66 [0.51, 0.85]
Li 2012	22	42	24	34	3.8%	0.46 [0.18, 1.19]
Xie 2015	11	124	39	230	5.2%	0.48 [0.23, 0.97]
Zhang 2014-C1	67	80	81	83	2.0%	0.13 [0.03, 0.58]
Zhang 2014-C2	136	162	288	317	6.1%	0.53 [0.30, 0.93]
**Subtotal (95% CI)**		**2737**		**3070**	**42.8%**	**0.60 [0.49, 0.74]**
Total events	1778		2189			
Heterogeneity: Tau² = 0.02; Chi² = 10.41, df = 7 (P = 0.17); I² = 33%						
Test for overall effect: Z = 4.82 (P < 0.00001)						
**Total (95% CI)**		**4566**		**5031**	**100.0%**	**0.49 [0.39, 0.63]**
Total events	2476		3302			
Heterogeneity: Tau² = 0.17; Chi² = 75.72, df = 17 (P < 0.00001); I² = 78%						
Test for overall effect: Z = 5.73 (P < 0.00001)						
Test for subgroup differences: Chi² = 1.14, df = 1 (P = 0.29), I² = 12.4%						

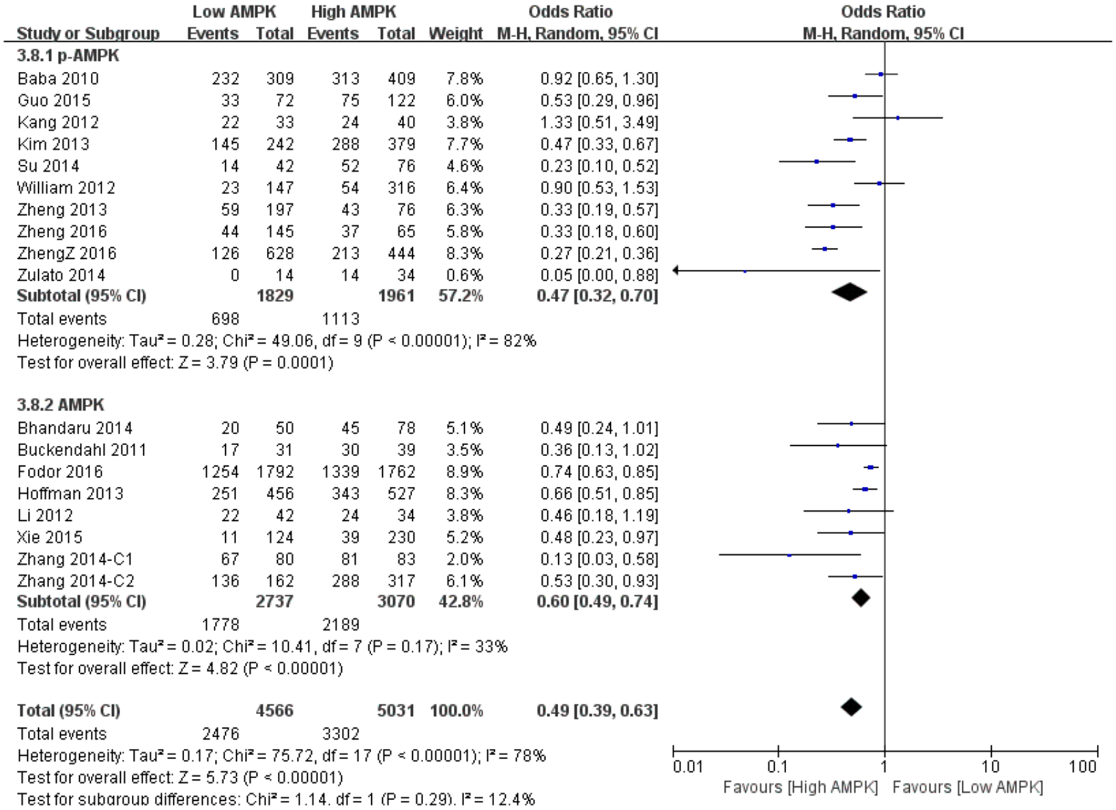

Supplementary Figure S11: The correlation between AMPK expression levels and 5-year overall survival among patients with different AMPK activation status.

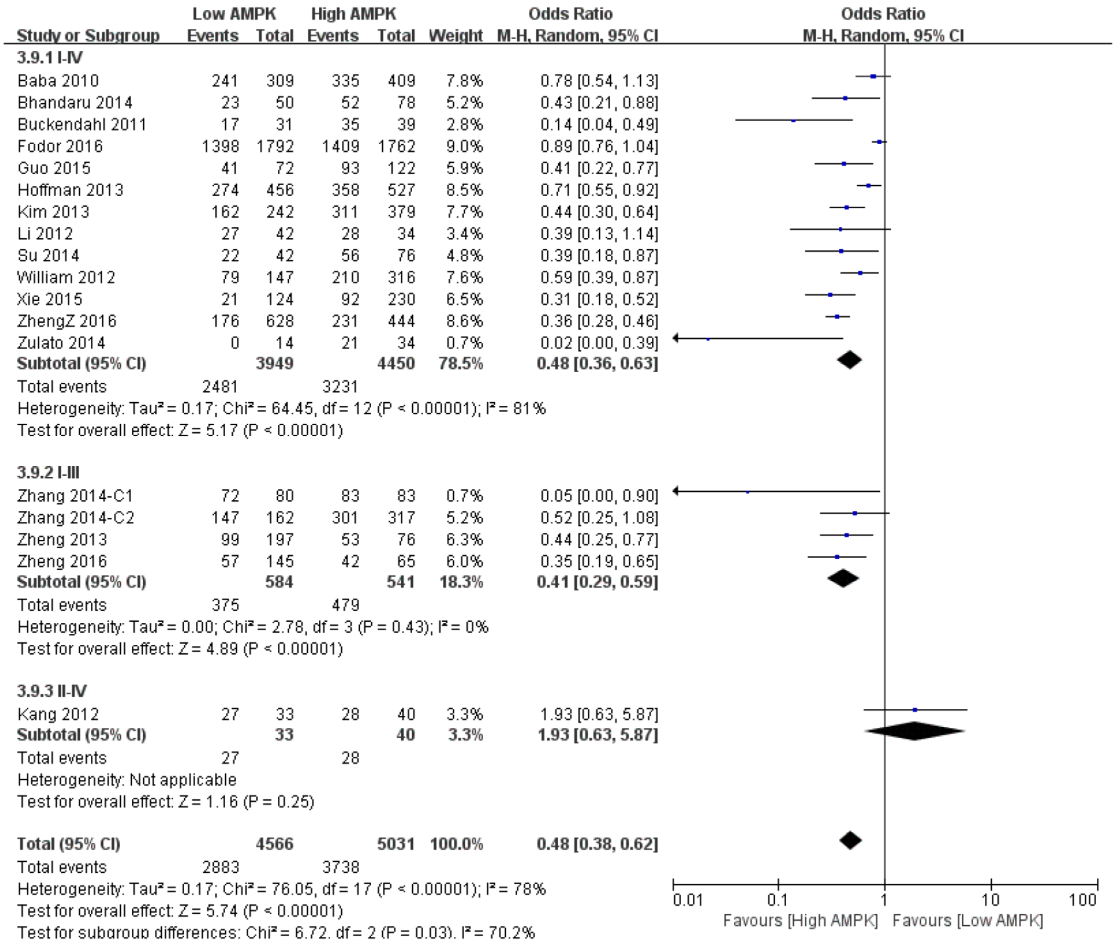

Supplementary Figure S12: The correlation between AMPK expression levels and 3-year overall survival among patients with different TNM stages.

Study or Subgroup	Low AMPK Events	Low AMPK Total	High AMPK Events	High AMPK Total	Weight	Odds Ratio M-H, Random, 95% CI
**3.10.1 I-IV**						
Baba 2010	232	309	313	409	7.8%	0.92 [0.65, 1.30]
Bhandaru 2014	20	50	45	78	5.1%	0.49 [0.24, 1.01]
Buckendahl 2011	17	31	30	39	3.5%	0.36 [0.13, 1.02]
Fodor 2016	1254	1792	1339	1762	8.9%	0.74 [0.63, 0.85]
Guo 2015	33	72	75	122	6.0%	0.53 [0.29, 0.96]
Hoffman 2013	251	456	343	527	8.3%	0.66 [0.51, 0.85]
Kim 2013	145	242	288	379	7.7%	0.47 [0.33, 0.67]
Li 2012	22	42	24	34	3.8%	0.46 [0.18, 1.19]
Su 2014	14	42	52	76	4.6%	0.23 [0.10, 0.52]
William 2012	23	147	54	316	6.4%	0.90 [0.53, 1.53]
Xie 2015	11	124	39	230	5.2%	0.48 [0.23, 0.97]
ZhengZ 2016	126	628	213	444	8.3%	0.27 [0.21, 0.36]
Zulato 2014	0	14	14	34	0.6%	0.05 [0.00, 0.88]
**Subtotal (95% CI)**		**3949**		**4450**	**76.0%**	**0.52 [0.39, 0.68]**
Total events	2148		2829			
Heterogeneity: Tau² = 0.16; Chi² = 61.16, df = 12 (P < 0.00001); I² = 80%						
Test for overall effect: Z = 4.72 (P < 0.00001)						
**3.10.2 I-III**						
Zhang 2014-C1	67	80	81	83	2.0%	0.13 [0.03, 0.58]
Zhang 2014-C2	136	162	288	317	6.1%	0.53 [0.30, 0.93]
Zheng 2013	59	197	43	76	6.3%	0.33 [0.19, 0.57]
Zheng 2016	44	145	37	65	5.8%	0.33 [0.18, 0.60]
**Subtotal (95% CI)**		**584**		**541**	**20.2%**	**0.36 [0.25, 0.52]**
Total events	306		449			
Heterogeneity: Tau² = 0.03; Chi² = 3.72, df = 3 (P = 0.29); I² = 19%						
Test for overall effect: Z = 5.38 (P < 0.00001)						
**3.10.3 II-IV**						
Kang 2012	22	33	24	40	3.8%	1.33 [0.51, 3.49]
**Subtotal (95% CI)**		**33**		**40**	**3.8%**	**1.33 [0.51, 3.49]**
Total events	22		24			
Heterogeneity: Not applicable						
Test for overall effect: Z = 0.59 (P = 0.56)						
**Total (95% CI)**		**4566**		**5031**	**100.0%**	**0.49 [0.39, 0.63]**
Total events	2476		3302			
Heterogeneity: Tau² = 0.17; Chi² = 75.72, df = 17 (P < 0.00001); I² = 78%						
Test for overall effect: Z = 5.73 (P < 0.00001)						
Test for subgroup differences: Chi² = 6.83, df = 2 (P = 0.03), I² = 70.7%						

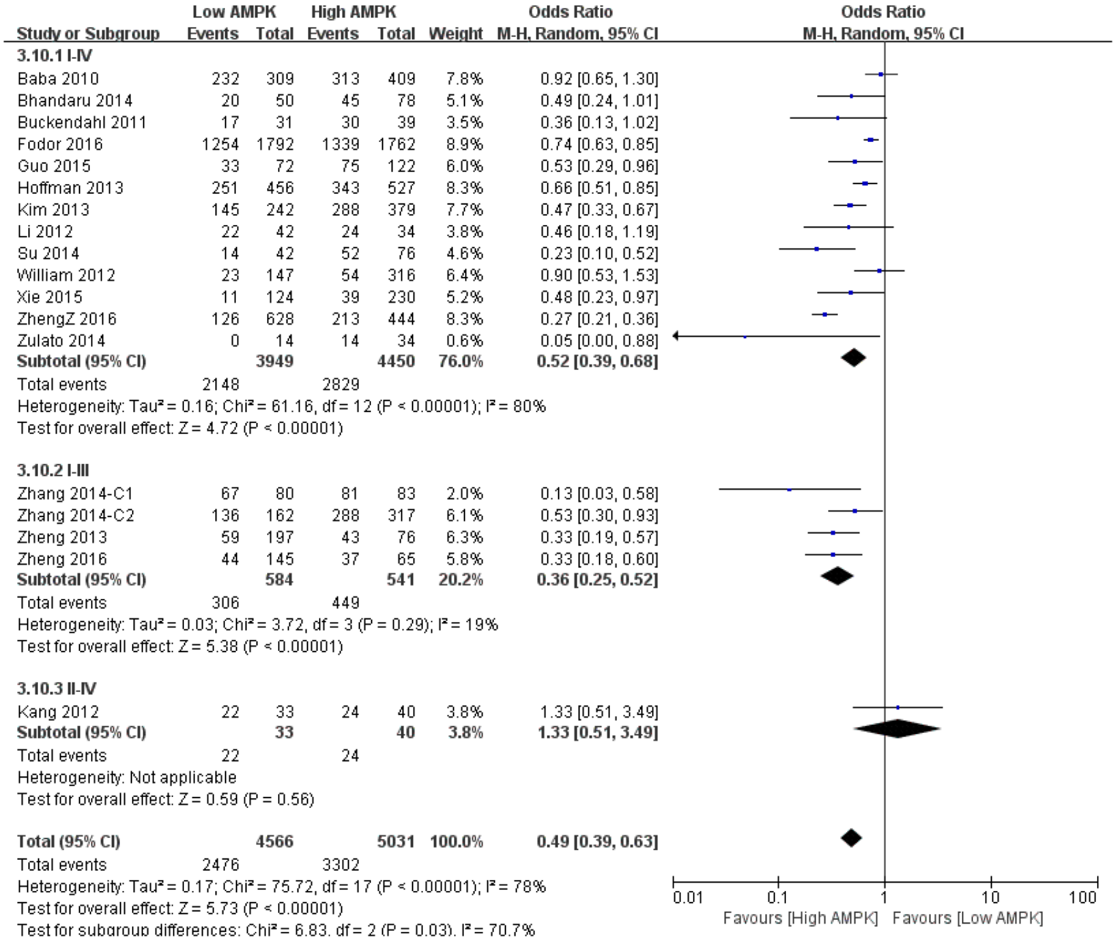

Supplementary Figure S13: The correlation between AMPK expression levels and 5-year overall survival among patients with different TNM stages.

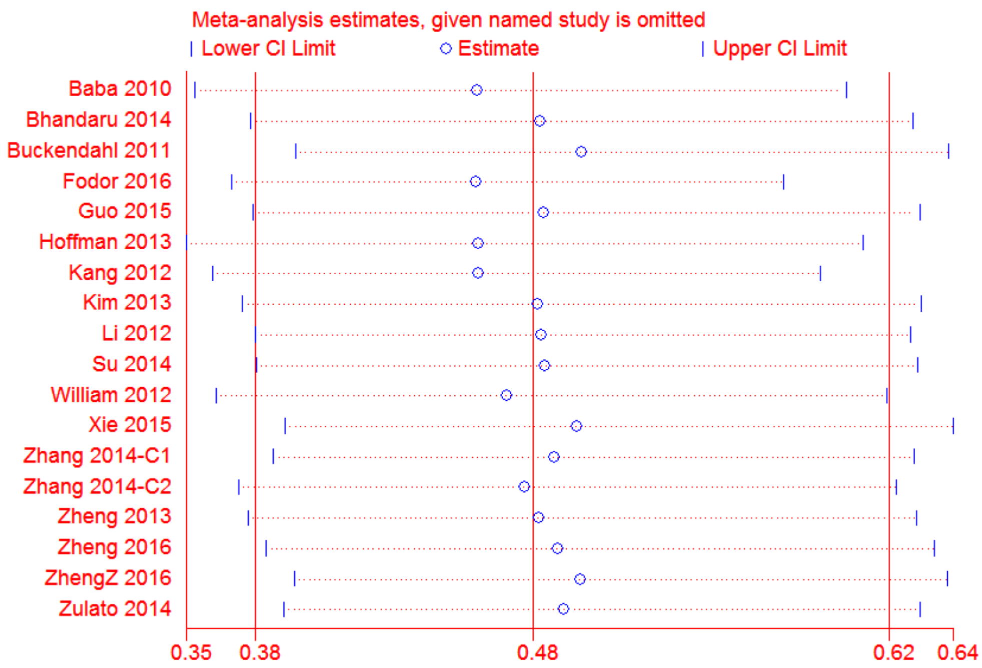

Supplementary Figure S14: Sensitivity analysis of 3-year overall survival.

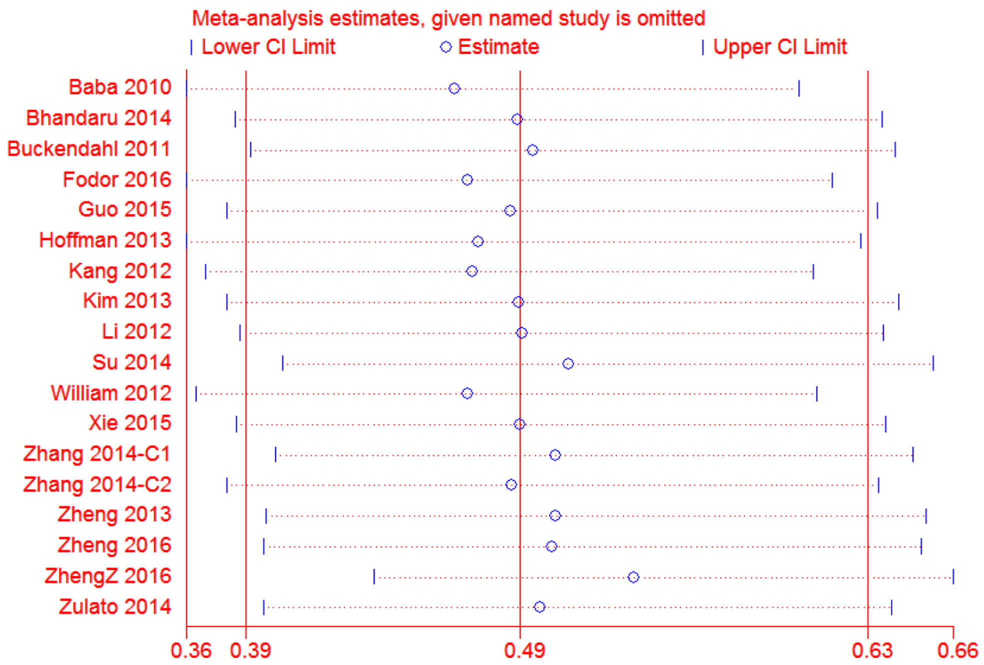

Supplementary Figure S15: Sensitivity analysis of 5-year overall survival.

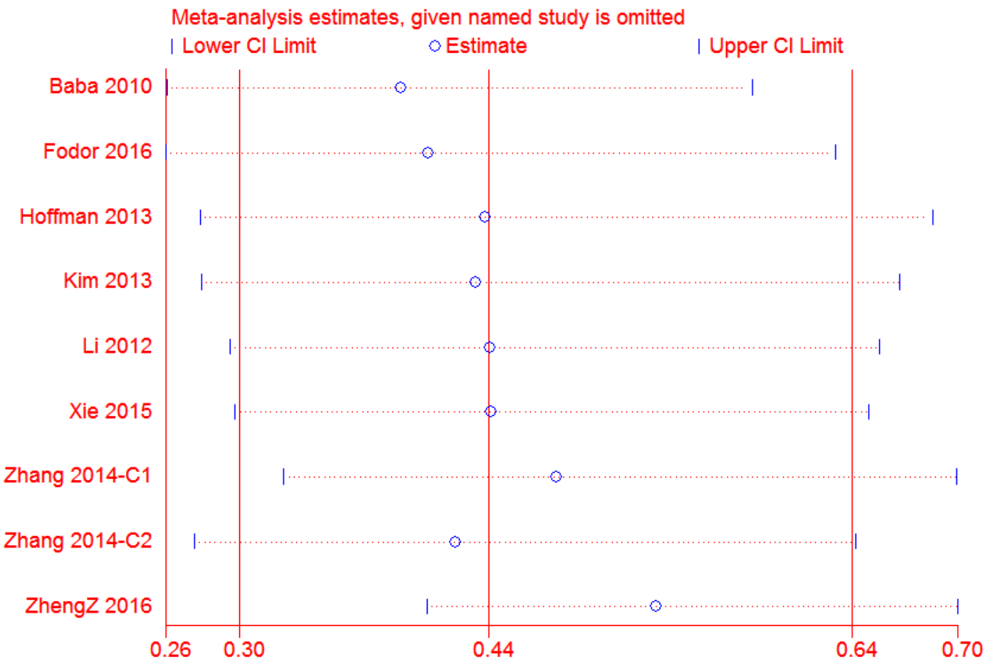

Supplementary Figure S16: Sensitivity analysis of 10-year overall survival.

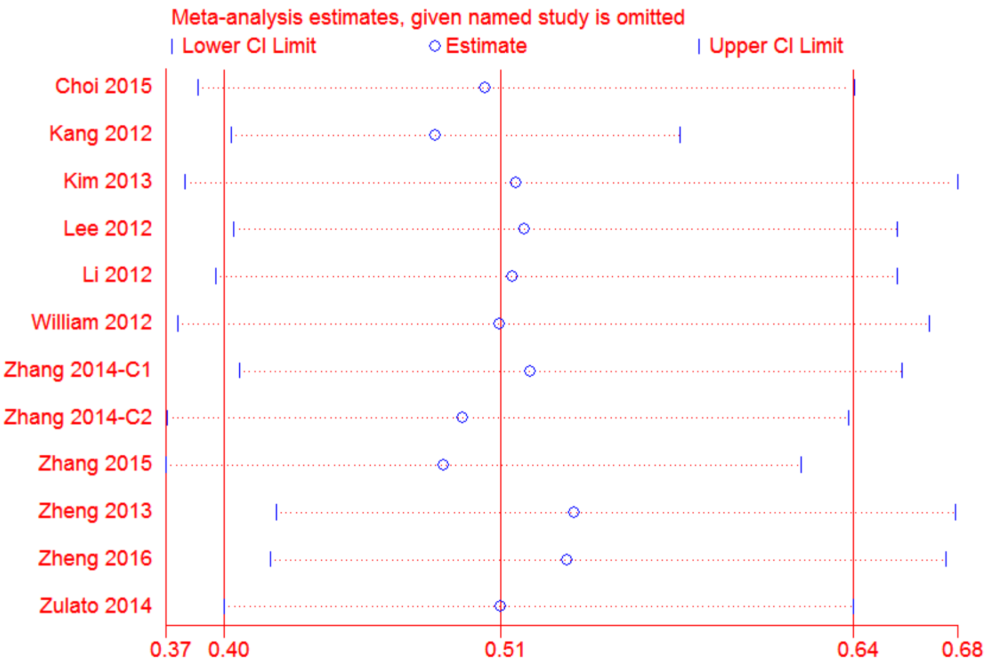

Supplementary Figure S17: Sensitivity analysis of 3-year disease free survival.

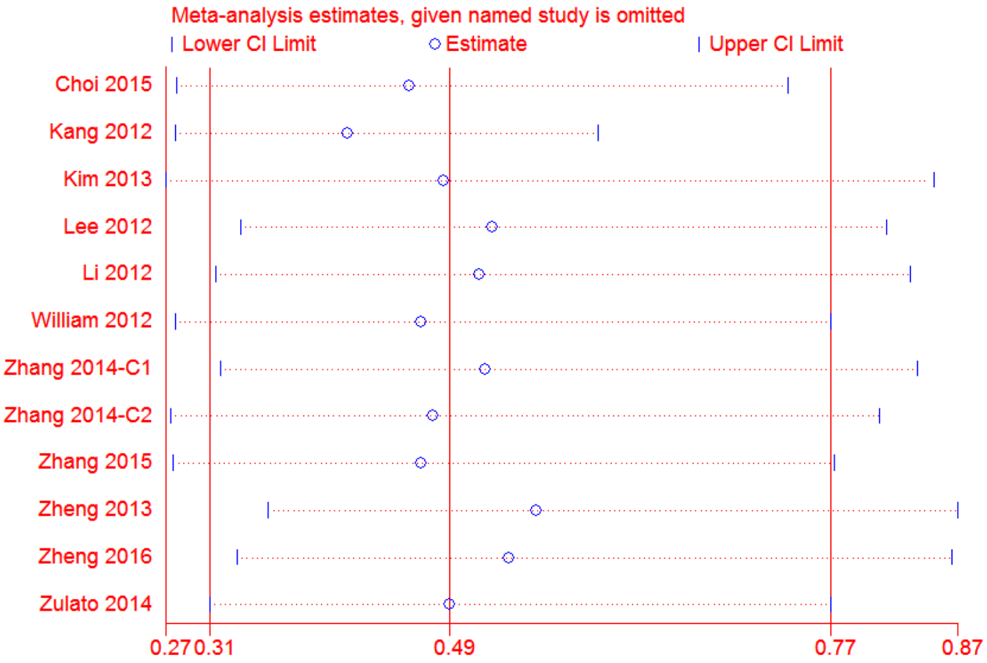

Supplementary Figure S18: Sensitivity analysis of 5-year disease free survival.

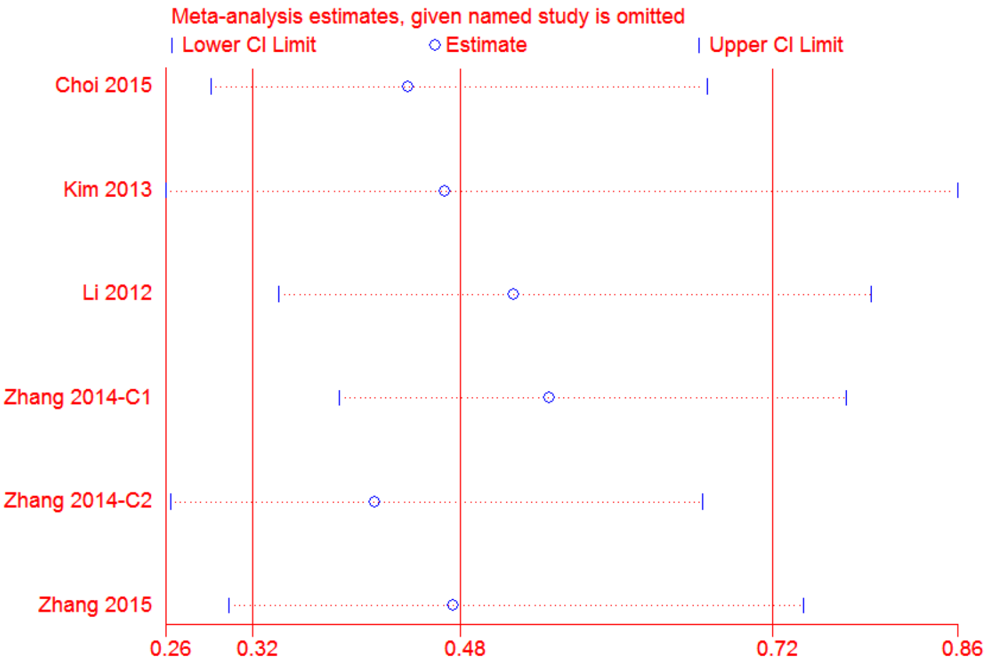

Supplementary Figure S19: Sensitivity analysis of 10-year disease free survival.

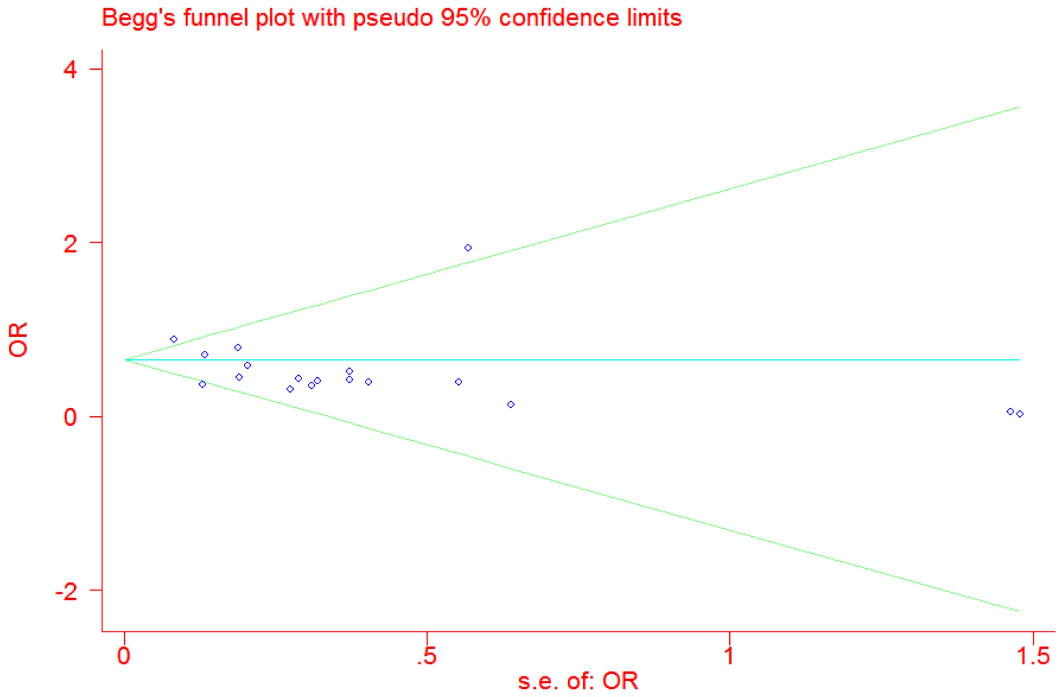

Supplementary Figure S20: Begg’s test of 3-year overall survival.

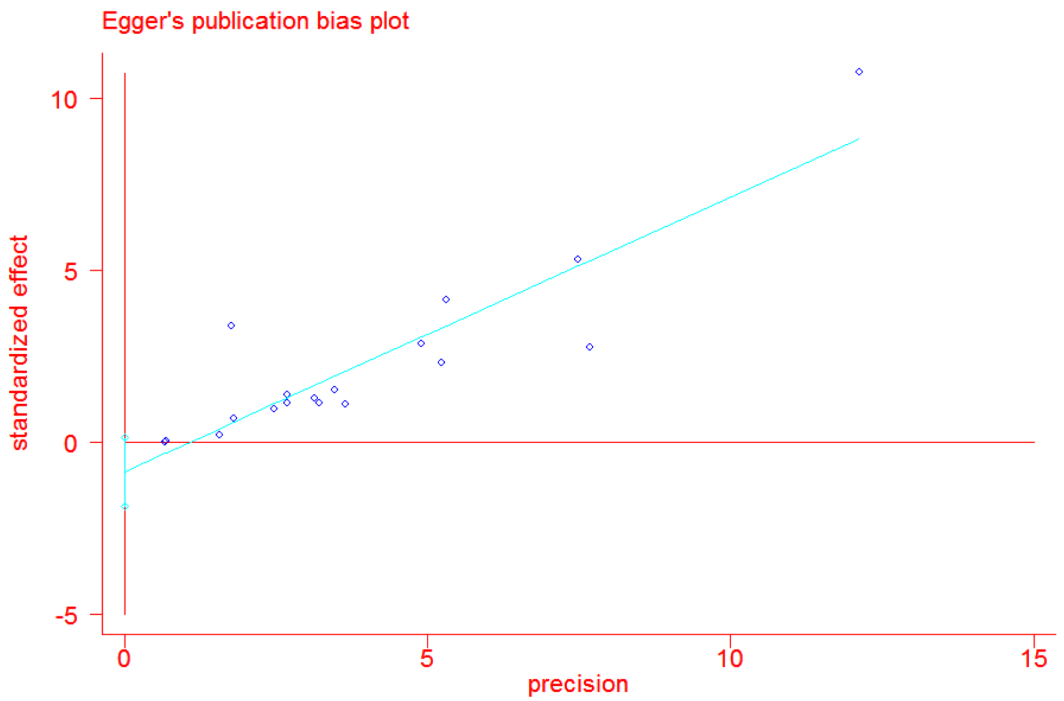

Supplementary Figure S21: Egger's test of 3-year overall survival.
